# Exosomes mediate intercellular transmission of Tembusu virus via the NS4A-Rab27a axis

**DOI:** 10.1186/s13567-026-01772-4

**Published:** 2026-08-09

**Authors:** Dalin He, Jing Yang, Yitong Cui, Bingrong Wu, Feng Wei, Siming Zhu, Youxiang Diao, Yi Tang

**Affiliations:** 1 College of Veterinary Medicine, Shandong Agricultural University, Tai’an, 271018 Shandong Province China; 2 Institute of Animal Sciences of Chinese Academy of Agricultural Sciences, Beijing, 10091 China; 3 College of Agricultural Science and Technology, Shandong Agriculture and Engineering University, Jinan, 250100 Shandong Province China; 4 Shandong Vocational Animal Science and Veterinary College, Weifang, 261061 Shandong Province China

**Keywords:** Exosomes, Tembusu virus, CavME, NS4A, Rab27a

## Abstract

Accumulating data support the role of exosomes from virus-infected cells (especially RNA viruses) in the selective encapsulation of viral proteins, genomic material, and even whole virions, thereby mediating intercellular communication events and contributing to viral transmission. Tembusu virus (TMUV), a single-stranded RNA virus, has posed a threat to global public health since its emergence in 2010. However, whether exosomes can mediate TMUV intercellular transmission remains obscure. Here, we show that exosomes derived from TMUV-infected cells (TMUV-exosomes) contained viral genomic RNA and partial viral proteins (C, prM, E, NS1, NS2B, NS4B, and NS5). TMUV-exosomes enter HEK293 cells primarily via a dynamin-dependent, cholesterol-sensitive caveolae-mediated (CavME) endocytosis pathway, thereby suppressing the subsequent expression of key antiviral immune genes to create a proviral microenvironment that supports productive TMUV infection. Remarkably, central to this exosomal hijacking mechanism, the viral NS4A protein and host Rab27a protein form a critical regulatory axis. Specifically, NS4A upregulates Rab27a expression to potentiate the release of exosomes encapsulating viral nucleic acids and proteomic components. Overall, our study provides a mechanistic model for understanding the role of exosomes in TMUV intercellular transmission and identifies the NS4A–Rab27a axis as a critical regulatory node. Disrupting this interaction may represent a potential therapeutic strategy for TMUV infection, warranting further investigation.

**Supplementary Information:**

The online version contains supplementary material available at 10.1186/s13567-026-01772-4.

## Introduction

Tembusu virus (TMUV), a contagious pathogen primarily affecting the duck farming industry, has incurred substantial economic losses in China since its 2010 emergence [1–3]. Recent studies confirm TMUV exhibits broad host tropism, demonstrating infectivity in multiple avian species and efficient replication across diverse mammalian cell lines (including HeLa, Vero, A549, BHK21, and SH-SY5Y) and mosquito cell lines (C6/36 and *Aedes albopictus*) [4–7]. Furthermore, intracerebral inoculation establishes pathogenicity in BALB/c and Kunming mouse models [8, 9]. Although no human disease has been documented, serological evidence reveals anti-TMUV antibodies and viral RNA in serum samples from duck industry workers [10], suggesting potential zoonotic transmission. TMUV possesses an enveloped virion containing a 10 990-nucleotide positive-sense single-stranded RNA genome. Its open reading frame encodes a polyprotein that undergoes proteolytic cleavage by host and viral proteases into three structural proteins (C, prM, and E) and seven nonstructural (NS) proteins (NS1, NS2A, NS2B, NS3, NS4A, NS4B, NS5) [11]. To control the disease, multiple vaccine platforms have been developed against TMUV, including live-attenuated [12], inactivated [13], and subunit formulations [14, 15]. Notwithstanding the commercialization of attenuated and inactivated vaccines, these interventions demonstrate suboptimal protective efficacy and fail to confer comprehensive immunity against TMUV infection. Neutralizing antibodies (NAbs) are the key mediators of protective immune response to TMUV after both infection and vaccination [16]. However, prior studies involving monoclonal antibodies targeting TMUV prM and E proteins revealed these antibodies failed to neutralize cellular infection effectively. This observation supports the existence of other mechanisms of TMUV dissemination and/or its evasion of NAbs.

Exosomes is a lipid bilayer membranous vesicle produced by living cells through a series of biological mechanisms including endocytosis-fusion exocytosis and excreted from the cell through active secretion [17]. Exosomes can facilitate intercellular communication by transporting intracellular proteins, lipids, and nucleic acids [18–20]. Studies have shown that exosomes are one of the tools used by viruses, especially RNA viruses, to selectively package viral proteins or nucleic acids to escape from immune surveillance, which is more conducive to the spread of viruses [21]. Given the unexplored role of TMUV-exosomes in long-distance viral transmission, comprehensive characterization is imperative.

This study demonstrates that TMUV infection significantly enhances exosomal secretion in HEK293 cells. Exosomes derived from TMUV-infected cells (TMUV-exosome) contained complete TMUV genomic RNA and multiple structural (C, prM, E) and nonstructural proteins (NS1, NS2B, NS4B, NS5). Crucially, by co-opting a dynamin- and cholesterol-dependent CavME endocytic pathway for entry, TMUV-exosomes not only gain access to HEK293 cells but also suppress host cytokine responses, ultimately fostering a proviral microenvironment that facilitates productive TMUV infection. Central to this exosomal hijacking mechanism, the TMUV NS4A protein upregulates Rab27a expression to potentiate the release of exosomes encapsulating viral genomic RNA and proteomic components. These viral cargo-laden vesicles subsequently mediate intercellular transmission of infectious material to distal cells, facilitating systemic dissemination of TMUV. Overall, these findings confirm that TMUV-exosomes can mediate cell infection and promote TMUV replication via CavME endocytic pathway, and identify a potential immune escape mechanism by which TMUV selectively encapsulates viral material via exosomes.

## Materials and methods

### Plasmids and reagents

The human *Rab27a* gene was amplified using Phanta Max Master Mix (Vazyme, China) with HEK293 cell-derived completmentary DNA (cDNA) as the template. The resulting polymerase chain reaction (PCR) product and the pCAGGS-HA vector were digested with *KpnI* and *NheI* restriction enzymes, followed by ligation to generate the pCAGGS–HA–Rab27a construct. The amplification primers were as follows: forward, 5′-TTCGAGCTCATCGATGGTACCATGTCTGATGGAGATT-3′, and reverse, 5′-TAATTAATTAAGATCTGCTAGCAGCCACATGCCCCTTT-3′. Plasmids encoding TMUV structural [capsid (C), prM, E] and nonstructural (NS1, NS2A, NS2B, NS3, NS4A, NS4B, NS5) proteins in the pCAGGS vector were constructed previously. All plasmid constructs were verified by DNA sequencing, and protein expression was confirmed by western blotting analysis. The chemical inhibitors of endocytic pathways used in this study and their sources are as follows: Dynamin (324410), Nystatin (475914), MβCD (332615), and NH_4_Cl (326372) were from Sigma-Aldrich.

### Cell and cell culture

Human embryonic kidney 293 (HEK293) cells were obtained commercially from the American Type Culture Collection (ATCC) and maintained in modified Eagle’s medium supplemented with 10% fetal bovine serum, penicillin (100 U/mL), and streptomycin (100 mg/mL) at 37 ℃ in an incubator with 5% CO_2_.

### Virus infection

TMUV-TC2B strain (GenBank: MH764605) were preserved by our laboratory [22]. For in vitro TMUV infection, cells were infected at an multiplicity of infection (MOI) of 1 and subsequently harvested at the indicated time points for analysis.

### Exosome isolation and purification

Exosomes were isolated from supernatants of TMUV-infected HEK293 cells using differential centrifugation. Briefly, the supernatant was sequentially centrifuged at 300 × *g* for 10 min to remove cells and at 2000 ×*g* for 10 min to pellet cellular debris. The resulting supernatant was then filtered through a 0.22 μm filter to eliminate large vesicles and concentrated using a 100 kDa ultrafiltration tube (Millipore, USA). The concentrate was subjected to ultracentrifugation at 140 000 × *g* for 70 min. The resulting pellet was resuspended in cold phosphate-buffered saline (PBS) and washed by repeating the ultracentrifugation step to remove contaminating proteins. The final pellet, containing purified exosomes, was resuspended in PBS for subsequent use.

### Western blotting

Western blotting was performed as previously described [23]. Briefly, protein samples of predetermined concentrations were separated by electrophoresis on 12% sodium dodecyl sulfate (SDS)–polyacrylamide gels. The resolved proteins were subsequently transferred onto 0.2 µm polyvinylidene difluoride (PVDF) membranes (Millipore, USA). Following transfer, the membranes were blocked for 1 h at room temperature with Tris-buffered saline containing 0.1% Tween-20 (TBST) and 10% nonfat dry milk. The membranes were then probed with specific primary antibodies at 4 °C overnight. After extensive washing, the blots were incubated for 1 h at room temperature with appropriate horseradish peroxidase (HRP)-conjugated anti-mouse or anti-rabbit secondary antibodies (Beyotime, China). Protein bands were finally visualized using the Clarity ECL Western Blotting Substrate (NCM Biotech, China) and imaged with a chemiluminescence detection system. The primary antibodies used in this study included those against CD63 (Abcam, UK), CD81 (Abcam, UK), TSG101 (Abcam, UK), Calnexin (Abcam, UK), *α*-Tubulin (Proteintech, China), Rab27a (Proteintech, China), and HA-tag (Abclonal, China), as well as TMUV prM, E, and NS5 proteins (made in our laboratory).

### Identification of TMUV RNA

After treating the exosomes from the TMUV-infection group with RNase H for 30 min, total RNA from exosomes was isolated from exosomes using a commercial total exosome RNA and protein isolation kit (Life Technologies, USA), following the manufacturer’s protocol. To ensure comprehensive coverage of the viral genome, the entire TMUV genome was amplified in 12 segments for the reverse transcription PCR. The specific primer sequences used for each fragment are provided in Additional file 1.

### LC–MS/MS

Purified exosomes were processed for liquid chromatography–tandem mass spectrometry (LC–MS/MS) analysis via the filtration-assisted sample preparation (FASP) method [24]. Proteins were solubilized in SDS–dithiothreitol (DTT)–Tris (SDT) buffer, and impurities were removed by ultrafiltration in urea (UA) buffer. After alkylation with iodoacetamide and subsequent washes, proteins were digested overnight with trypsin. The resulting peptides were analyzed by LC–MS/MS on a Q Exactive mass spectrometer coupled to an Easy-nLC 1200 system.

### Exosome infected cell assay

The purified TMUV-derived exosomes were pre-incubated with a high concentration of TMUV-specific neutralizing antibodies (directed against the E protein) for 1 h at 37 °C prior to inoculation into naive HEK293 cells. HEK293 cells were plated in 24-well plates at a density of 2 × 10^5^ cells per well. The cells were then exposed to exosomes derived from TMUV-infected cell cultures for 2 h at 37 °C under 5% CO_2_. Following exposure, the medium was replaced with fresh maintenance medium. At the indicated time points (12, 24, 36, and 48 h), cells were either fixed for indirect immunofluorescence assay or collected for western blotting analysis.

### Indirect immunofluorescence assay (IFA)

Cells were fixed with 4% paraformaldehyde for 20 min, permeabilized with 0.1% Triton X-100 for 10 min and blocked with 5% bovine serum albumin (BSA; Sigma-Aldrich) for 30 min. Subsequently, the cells were incubated with specific primary antibodies for 3 h, followed by incubation with Alexa Fluor 594- or 488-conjugated secondary antibodies (Proteintech, China) for 90 min. Nuclei were counterstained with 4′,6-diamidino-2-phenylindole (DAPI) (Beyotime, China). Fluorescent images were captured using a laser scanning confocal microscope (Leica, Germany) equipped with LAS X software.

### Real-time quantitative polymerase chain reaction (qPCR)

Total RNA was isolated from cell samples using the MiniBEST Universal RNA Extraction Kit (TaKaRa, Japan) according to the manufacturer’s instructions. Subsequent cDNA synthesis was performed using a one-step SYBR RT-PCR kit (TaKaRa, Japan). qPCR was then conducted on a QuantStudio 6 system (Applied Biosystems, USA) with the UltraSYBR mixture (CWBIO, China). All primer sequences used are listed in Additional file 2. The average threshold cycle (Ct) values from technical triplicates were normalized to the housekeeping gene *GAPDH*.

### Statistical analysis

Statistical analyses were performed using GraphPad Prism software (version 9.0, USA). Data are presented as the mean ± standard deviation (SD). Differences between groups were assessed by an unpaired, two-tailed Student’s *t*-test. A *P*-value of less than 0.05 (*P* < 0.05) or 0.01 (*P* < 0.01) was considered statistically significant.

## Results

### Isolation and characterization of exosomes derived from TMUV-infected cells

**Figure 1:**
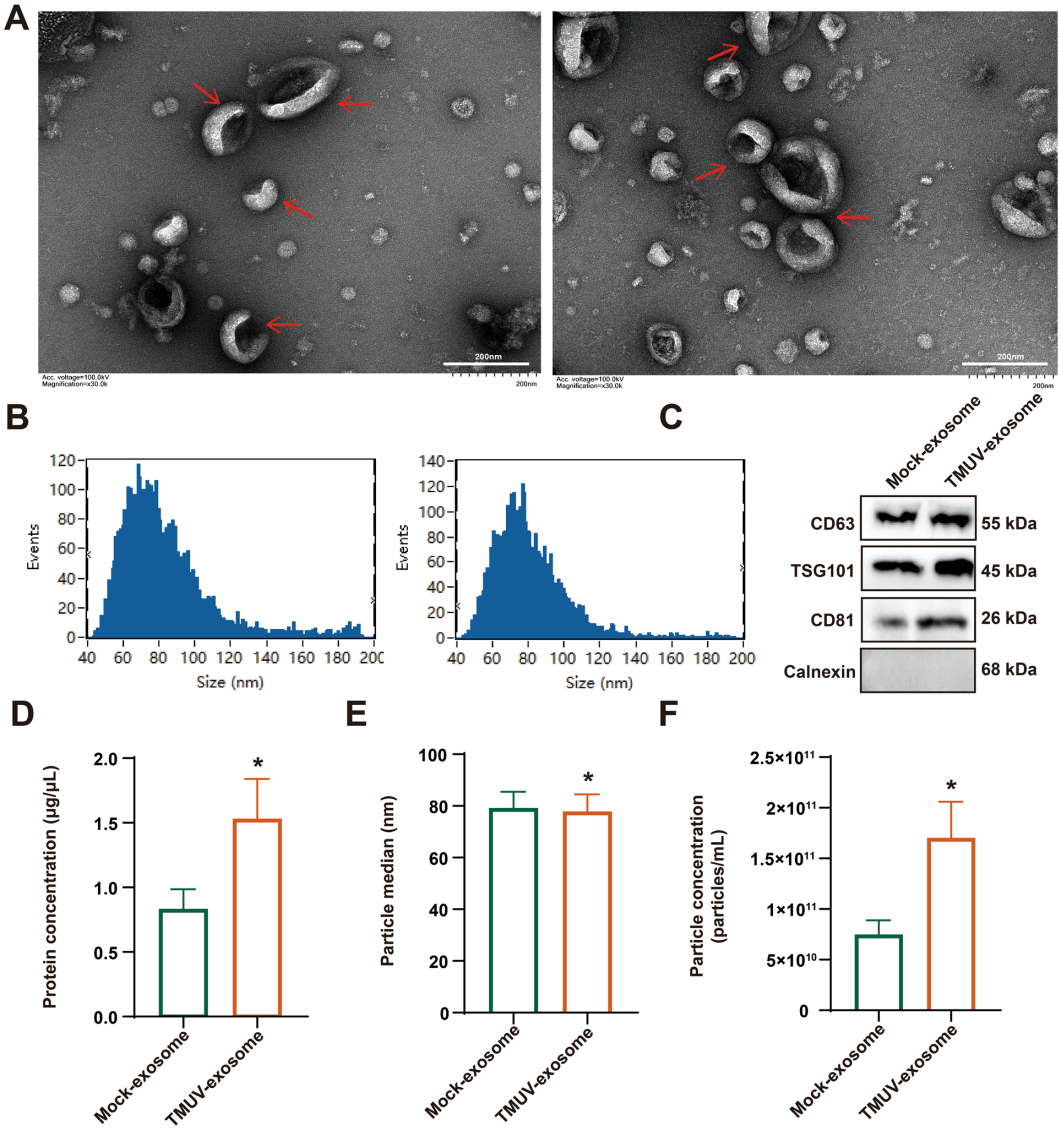
**Purification and profiling of exosomes derived from Mock- and TMUV-infected cells. A** Representative transmission electron microscopy (TEM) images of negatively stained exosomes. Exosomes were purified from the supernatants of Mock- (left panel) and TMUV-infected (right panel) HEK293 cells. **B** Nano flow cytometry (NanoFCM) was performed to determine the size distribution of exosomes purified from Mock- (left) and TMUV-infected (right) HEK293 cells. **C** Purified exosomes derived from Mock- or TMUV-infected cells were probed with antibodies against the exosomal markers CD63, TSG101, and CD81, as well as the endoplasmic reticulum resident protein Calnexin (negative control). **D** Quantitative analysis of total exosomal protein yield from Mock- versus TMUV-infected HEK293 cells. **E** Comparative analysis of exosomal hydrodynamic diameter, as determined by NanoFCM. **F** Comparison of exosomal particle concentration between Mock- and TMUV-infected cells, quantified by NanoFCM.

Due to the overlapping physical properties (e.g., size and buoyant density) of TMUV virions and exosomes, conventional ultracentrifugation alone results in significant viral co-isolation. To overcome this limitation and obtain exosomes of high purity from infected cells, we implemented a stringent protocol combining ultrafiltration concentration with sequential ultracentrifugation and CD63-specific immunomagnetic bead affinity purification. To this end, HEK293 cells were infected with the TMUV strain TC2B at a multiplicity of infection (MOI) of 1.0. Following infection, cells were maintained in Dulbecco’s modified Eagle medium (DMEM) supplemented with 2% exosome-depleted fetal bovine serum (FBS). At 24 h postinfection (hpi), culture supernatants were collected for the subsequent isolation and purification of exosomes. The purified exosomes were characterized using nanoflow cytometry, transmission electron microscopy (TEM), and western blotting analysis for exosomal markers. TEM analysis revealed that the purified exosomes exhibited a characteristic cup-shaped morphology with a central depression and raised edges, demonstrating good structural integrity and high purity. No viral particles were observed (Figure 1A). Nanoflow cytometry analysis of the purified exosomes revealed a particle size distribution ranging from 30 to 200 nm (Figure 1B). Three representative exosomal markers, namely CD63, TSG101, and CD81, were detected in the purified exosomes by western blotting (Figure 1C). Furthermore, TMUV infection led to a comprehensive change in the characteristics of cell-derived exosomes, manifesting as an increase in exosomal protein concentration (Figure 1D), a decrease in particle size (Figure 1E), and an increase in particle concentration (Figure 1F).

### TMUV-exosomes derived from TMUV-infected cells contain viral components and host proteins

**Figure 2:**
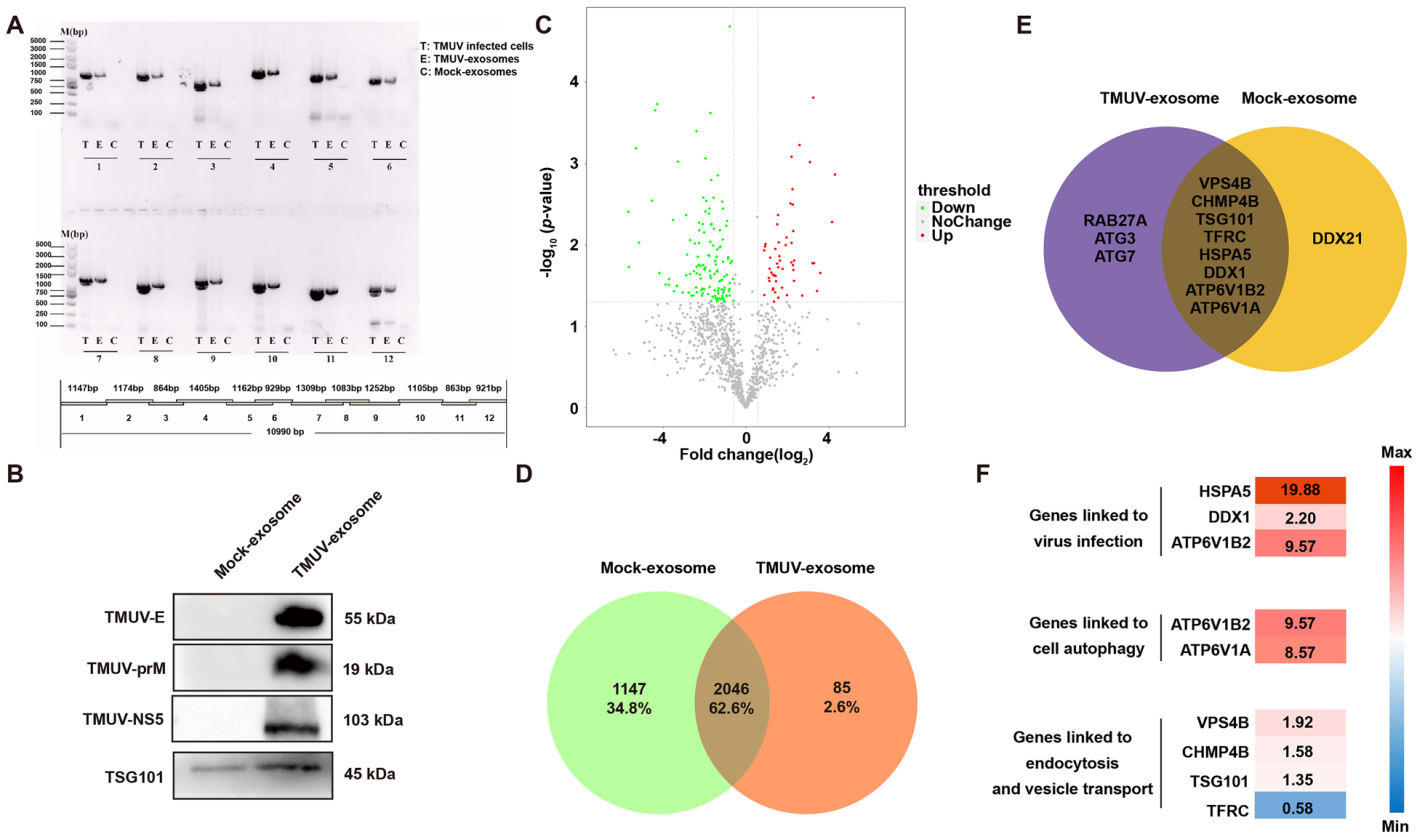
**Molecular characterization of exosomal components derived from TMUV-infected HEK293 cells**. **A** RT-PCR analysis was performed to assay for the presence of TMUV genomic RNA in exosomes isolated from infected HEK293 cells. **B** Immunoblotting analysis of exosomal lysates using antibodies against the viral E, prM, and NS5 proteins. TSG101 was used as an exosomal loading control. **C** Volcano plot displaying proteins with significant abundance changes in exosomes from TMUV-infected versus Mock-infected HEK293 cells. Dashed lines indicate thresholds for statistical significance and fold-change. **D** Venn diagram showing the number of proteins identified in exosomes from Mock- and TMUV-infected cells, as well as those common to both groups. **E** Venn diagram illustrating the overlap among significantly upregulated or downregulated proteins in exosomes from Mock- versus TMUV-infected HEK293 cells. **F** Heatmap-style bar plot showing the fold-change and functional categorization of key exosomal proteins altered by TMUV infection. Red and blue bars represent up- and down-regulated proteins, respectively.

To characterize the molecular composition of exosomes derived from TMUV-infected cells, we analyzed their nucleic acid and protein contents. Real-time PCR (RT-PCR) confirmed the presence of the full-length TMUV genome (Figure 2A), while proteomic profiling revealed a selective enrichment of viral proteins—specifically capsid (C), prM, E, NS1, NS2B, NS4B, and NS5—but not NS2A, NS3, or NS4A (data not shown). The incorporation of E, prM, and NS5 proteins was further validated by western blotting using in-house-generated monoclonal antibodies (Figure 2B).

Using a liquid chromatography–tandem mass spectrometry (LC–MS/MS), we identified a total of 5360 host proteins in TMUV- and Mock-exosomes, among which 1106 were differentially expressed: 52 up-regulated and 130 down-regulated (Figure 2C, D). These proteins are implicated in key cellular processes such as endocytosis, vesicular transport, viral infection, and autophagy, indicating that TMUV infection reshapes the host proteome and directs its selective packaging into exosomes. Notably, several proteins involved in exosome biogenesis and release—including CHMP4B, VPS4B, and TSG101—were significantly up-regulated, while Rab27a was exclusively detected in infected cells, consistent with enhanced exosome secretion during TMUV infection (Figures 1C and 2B). Additionally, multiple factors facilitating viral entry and replication, such as HSPA5, DDX1, and ATP6V1B2, were up-regulated. Among these, ATP6V1B2, a component of the vacuolar proton pump, contributes to endosomal acidification and viral replication. Autophagy-related proteins including ATP6V1B2, ATP6V1A, ATG3, and ATG7 were also markedly up-regulated or uniquely present in TMUV-exosomes (Figure 2E, F). Taken together, these findings demonstrate that TMUV infection reprograms the host cell proteome and promotes the selective packaging of specific viral and host factors into exosomes, which may modulate recipient cell processes and facilitate viral propagation.

### TMUV-exosomes internalized by HEK293 cells undergo replication

**Figure 3:**
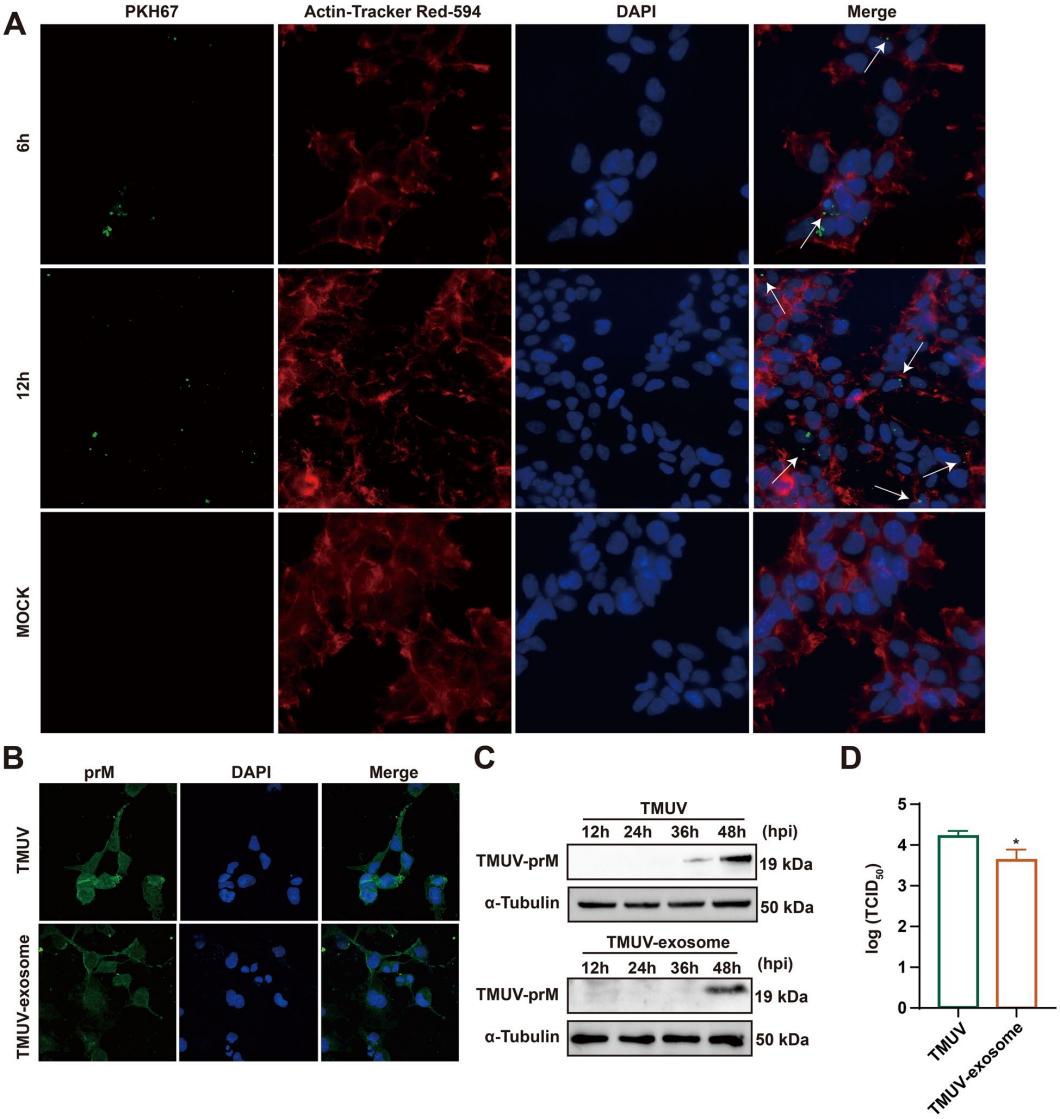
**TMUV-exosomes internalization and propagation in HEK293 cells**. **A** Cellular internalization of PKH67-labeled TMUV-exosomes observed by CLSM. TMUV-exosomes (green), actin filaments (red), and cell nucleus (blue). **B** Detection of productive TMUV infection via indirect immunofluorescence assay (IFA) in HEK293 cells inoculated with TMUV-exosomes. **C** Immunoblotting analysis of TMUV prM expression in HEK293 cells infected with TMUV or treated with TMUV-exosomes. **D** Viral titers determined by TCID_50_ assay in HEK293 cells infected with cell-derived TMUV or inoculated with TMUV-exosomes.

Having established the presence of TMUV components within purified exosomes, we next sought to determine whether these exosomes could mediate infection of naive cells. To assess cellular uptake, HEK293 cells were incubated with PKH67-labeled TMUV-exosomes. Cells were fixed at 6 and 12 hpi, stained with a microfilament probe, and visualized by confocal laser scanning microscope (CLSM). Cytosolic green fluorescence, indicative of internalized exosomes, was detected in a time-dependent manner, confirming exosome uptake (Figure 3A). In contrast, control cells showed only microfilament staining (red), confirming the specificity of the observed uptake. To determine the infectivity of exosomes derived from TMUV-infected cells, naive HEK293 cells were incubated with the purified exosomes. At 48 hpi, infection was confirmed by indirect immunofluorescence assay (IFA) using a monoclonal antibody against the TMUV PrM protein. Cells infected with cell-culture-derived TMUV served as a positive control. As shown in Figure 3B, both TMUV-exosomes and TMUV induced obvious cytopathic effects. Furthermore, IFA detection of the PrM protein confirmed that a productive infection was established in cells inoculated with TMUV-exosome, demonstrating their capacity to transmit the virus. The establishment of a productive infection in HEK293 cells inoculated with TMUV-exosomes was further confirmed by western blotting analysis, which detected the expression of the viral prM protein (Figure 3C). Corroborating this finding, titers of infectious virus were quantified by a 50% tissue culture infective dose (TCID_50_) assay. The results demonstrated that TMUV-exosomes generated viral titers in HEK293 cells that were comparable to those produced by cell-culture-derived TMUV, serving as a positive control (Figure 3D). Collectively, these data establish that TMUV-exosomes derived from TMUV-infected cells are fully competent for establishing a productive infection in naive HEK293 cells.

### Role of caveolae-mediated endocytosis (CavME) pathways in TMUV-exosomes internalization

**Figure 4:**
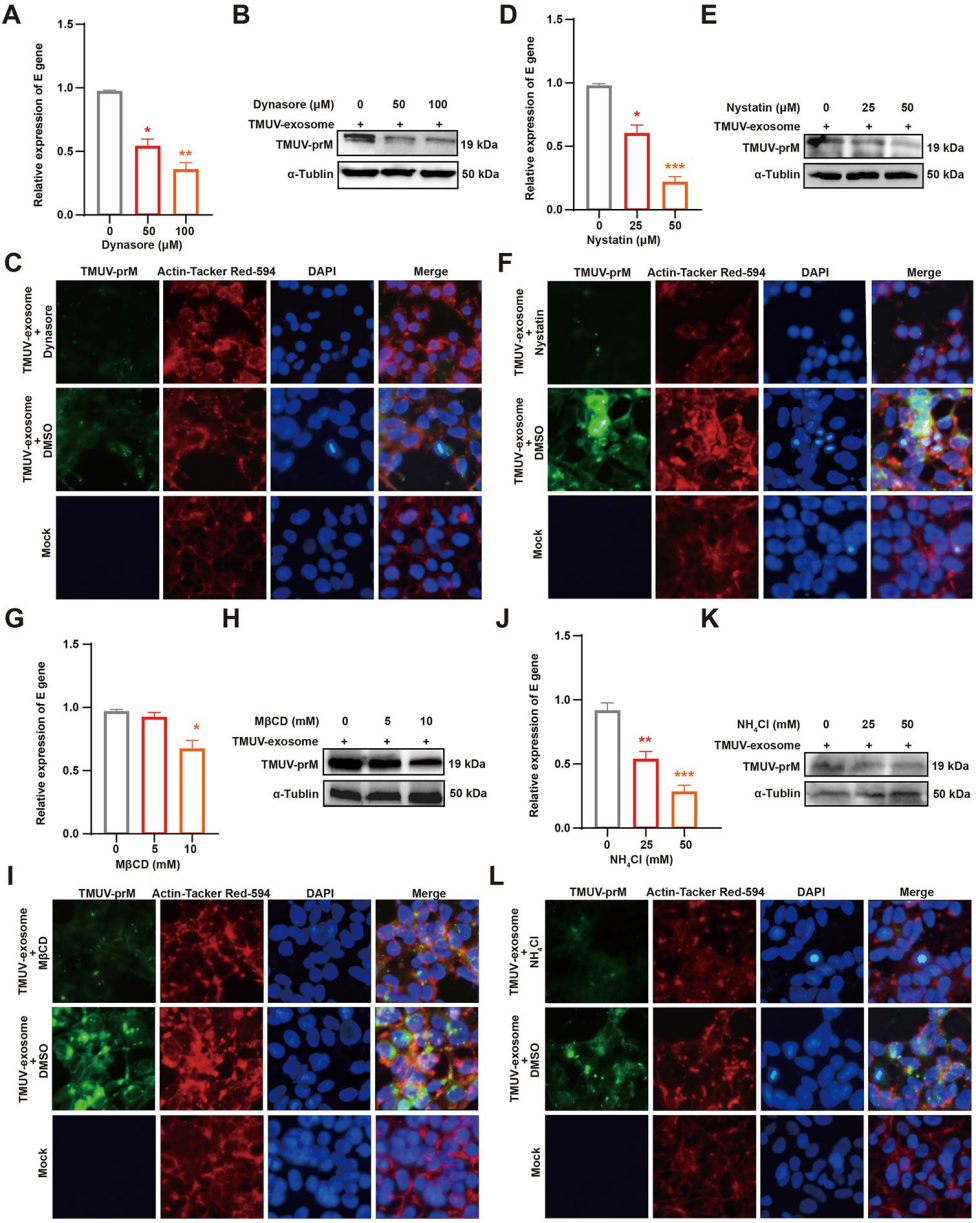
**CavME pathways involved in TMUV-exosomes entry into HEK293 cells**. **A** qPCR analysis of TMUV E gene expression in HEK293 cells infected with TMUV-exosomes for 24 h following treatment with Dynamin for 1 h. **B** Immunoblotting analysis of TMUV prM protein levels in HEK293 cells infected with TMUV-exosomes for 24 h following treatment with Dynamin for 1 h. **C** CLSM analysis showed that TMUV-exosomes entry was markedly inhibited in Dynamin-treated HEK293 cells. Actin filaments (red), prM protein (green), and cell nucleus (blue). **D** qPCR analysis of TMUV E gene expression in HEK293 cells infected with TMUV-exosomes for 24 h following treatment with Nystatin for 1 h. **E** Immunoblotting analysis of TMUV prM protein levels in HEK293 cells infected with TMUV-exosomes for 24 h following treatment with Nystatin for 1 h. **F** CLSM analysis showed that TMUV-exosomes entry was markedly inhibited in Nystatin-treated HEK293 cells. Actin filaments (red), prM protein (green), and cell nucleus (blue). **G** qPCR analysis of TMUV E gene expression in HEK293 cells infected with TMUV-exosomes for 24 h following treatment with MβCD for 1 h. **H** Immunoblotting analysis of TMUV prM protein levels in HEK293 cells infected with TMUV-exosomes for 24 h following treatment with MβCD for 1 h. **I** CLSM analysis showed that TMUV-exosomes entry was markedly inhibited in MβCD-treated HEK293 cells. Actin filaments (red), prM protein (green), and cell nucleus (blue). **J** qPCR analysis of TMUV E gene expression in HEK293 cells infected with TMUV-exosomes for 24 h following treatment with NH^4^Cl for 1 h. **K** Immunoblotting analysis of TMUV prM protein levels in HEK293 cells infected with TMUV-exosomes for 24 h following treatment with NH_4_Cl for 1 h. **L** CLSM analysis showed that TMUV-exosomes entry was markedly inhibited in NH_4_Cl-treated HEK293 cells. Actin filaments (red), prM protein (green), and cell nucleus (blue).

The GTPase dynamin, which mediates the scission of newly formed endocytic vesicles from the plasma membrane, is a key regulator of caveolae-mediated endocytosis (CavME) [25]. To investigate the role of dynamin in TMUV-exosome infection, HEK293 cells were treated with Dynasore, a reversible dynamin inhibitor [26]. RT-qPCR analysis showed that Dynasore reduced viral RNA levels in a dose-dependent manner (Figure 4A). Consistent with this, western blot analysis revealed that Dynasore treatment also suppressed infection in a dose-dependent fashion, as evidenced by decreased viral protein expression (Figure 4B). Furthermore, CLSM confirmed that Dynasore significantly impaired the internalization of TMUV-exosomes and abolished the formation of viral replication factories in treated cells (Figure 4C). Collectively, these results demonstrate that both the entry of TMUV-exosomes and subsequent post-entry steps in HEK293 cells are dynamin-dependent processes. To evaluate the role of CavME in TMUV-exosome entry, we examined the effect of nystatin, a specific CavME inhibitor, on this process. RT-qPCR assay indicated that Nystatin inhibited TMUV-exosomes propagation (Figure 4D). Western blotting then demonstrated that pretreating HEK293 cells with Nystatin significantly decreased viral protein expression (Figure 4E). Consistent with this, CLSM confirmed that Dynasore markedly impaired TMUV-exosome internalization and disrupted the formation of viral replication factories in HEK293 cells (Figure 4F). Together, these results demonstrate that TMUV-exosome entry into HEK293 cells occurs primarily through the CavME pathway.

Previous studies have indicated that viral entry occurs via the CavME pathway, a process strictly dependent on cholesterol, which constitutes a principal component of lipid rafts within caveolae [27]. To evaluate the role of CavME in the internalization of TMUV-exosomes into HEK293 cells, we tested the effect of methyl-β-cyclodextrin (MβCD), an agent that depletes cholesterol from the plasma membrane. The results demonstrated that the entry of TMUV-exosomes into cells via the CavME pathway requires cholesterol (Figure 4G–I). Furthermore, to investigate whether the cellular entry of TMUV-exosomes is pH-dependent, we applied ammonium chloride (_NH4_Cl), an inhibitor of endosomal acidification, to examine its effect on TMUV replication. The results demonstrated that the internalization of TMUV-exosomes via the CavME pathway occurs in a pH-dependent manner, indicating the requirement of an acidic environment for productive infection (Figure 4J–L).

### TMUV-exosomes promotes the replication of TMUV

**Figure 5:**
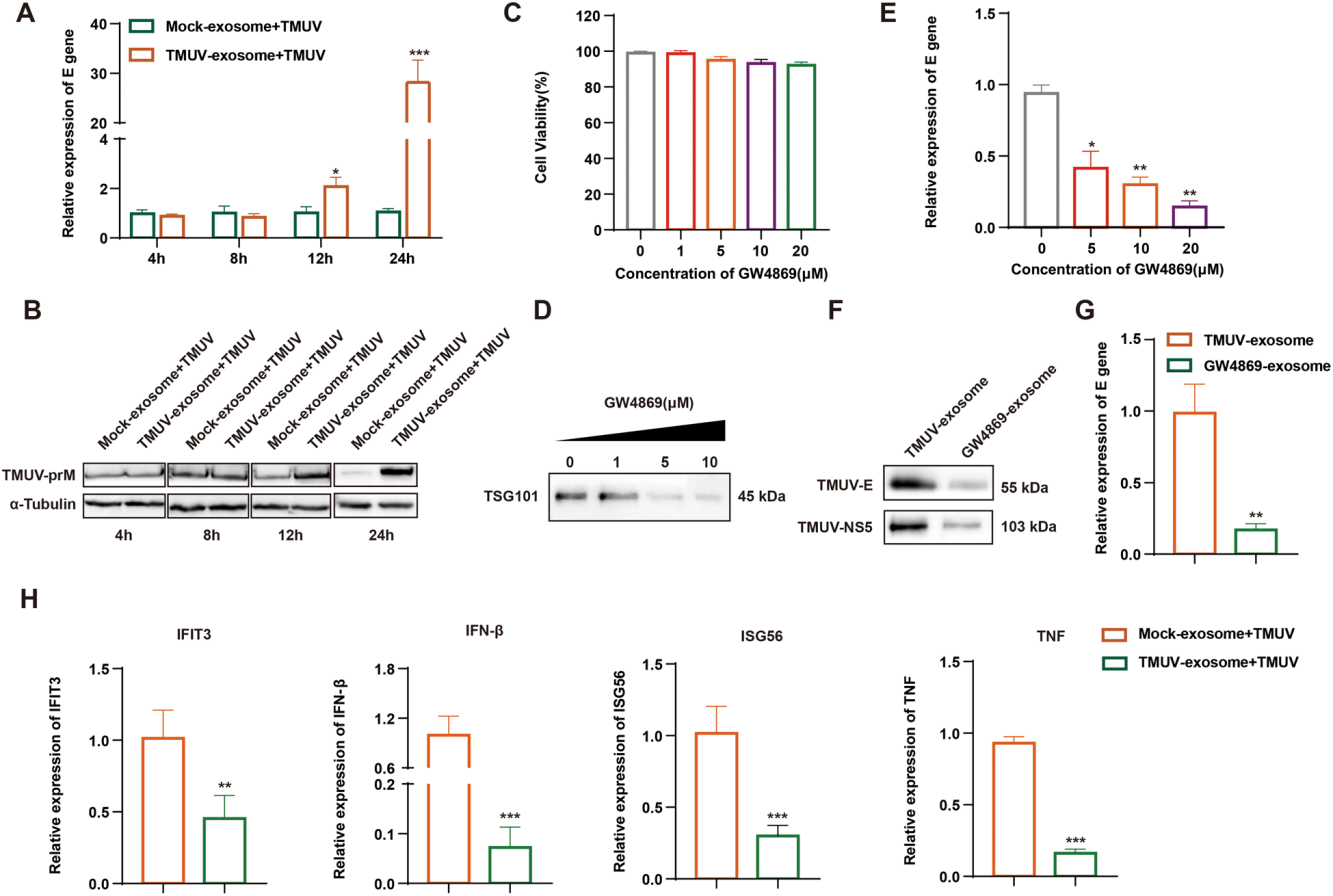
**TMUV-exosomes promote TMUV replication and modulate host immune response**. **A** qPCR analysis of TMUV E gene expression in HEK293 cells treated with exosomes isolated from Mock- or TMUV-infected cells at indicated time points (4, 8, 12, and 24 hpi). **B** Immunoblotting analysis of TMUV prM protein expression in cells incubated with exosomes collected at different time points from infection groups. **C** Cytotoxicity assessment of GW4869 in HEK293 cells at varying concentrations by MTT assay. **D** TSG101 expression in exosomes isolated from GW4869-treated HEK293 cells, analyzed by immunoblotting. **E** TMUV E gene transcription levels in infected HEK293 cells after GW4869 treatment at different concentrations. **F** Packaging efficiency of viral E and NS5 proteins in TMUV-exosomes from GW4869-treated cells, detected by immunoblotting. **G** Infectivity of TMUV-exosomes derived from GW4869-treated cells, evaluated by TMUV E gene expression in recipient cells. **H** Transcript levels of IFN-β, IFIT-3, ISG56, and TNF in HEK293 cells following TMUV infection (24 hpi) after pretreatment with TMUV-exosomes or Mock-exosomes.

Given that TMUV infection increases exosome secretion in HEK293 cells, we further investigated the role of the exosomal pathway in viral dissemination by examining its impact on TMUV replication. We first purified exosomes from TMUV-infected and Mock-infected cells at 4, 8, 12, and 24 hpi. HEK293 cells were pretreated with equal protein amounts of these exosomes and subsequently infected with TMUV. Viral replication was then assessed by measuring the relative expression of the TMUV E gene via RT-qPCR. The results showed that exosomes collected at 4 and 8 hpi had no significant effect on TMUV replication compared to the control. In contrast, exosomes harvested at 12 and 24 hpi significantly promoted viral replication, with the enhancing effect becoming more pronounced over time (Figure 5A). This time-dependent promotion was further confirmed at the protein level by western blotting analysis of TMUV prM expression, the trend of which was consistent with the transcriptional data (Figure 5B).

To determine whether inhibiting exosome release would impair exosome-mediated TMUV transmission, we examined the effect of the exosome release inhibitor GW4869 on TMUV replication. We first confirmed that GW4869 exhibited no apparent cytotoxicity in HEK293 cells across the tested concentration range (Figure 5C). To validate its efficacy in inhibiting exosome biogenesis, we monitored the expression of the exosomal marker TSG101 by western blotting. The results showed a marked, concentration-dependent reduction in TSG101 levels, indicating a progressive decrease in exosome production (Figure 5D). We next asked whether this exosome inhibition would affect TMUV replication. HEK293 cells were infected with TMUV and then treated with the indicated concentrations of GW4869. Subsequent RT-qPCR analysis revealed that GW4869 treatment significantly suppressed intracellular TMUV replication in a dose-dependent manner compared with the Mock-treated control (Figure 5E). We next investigated whether GW4869 affects the packaging of viral proteins into exosomes and, consequently, their infectivity. Western blotting analysis revealed that exosomes derived from GW4869-treated, infected cells (GW4869-exosomes) incorporated significantly less viral E and NS5 proteins than those from the untreated control (TMUV-exosomes) (Figure 5F). To functionally assess this change, naive HEK293 cells were inoculated with equal amounts of exosomes from both groups. RT-qPCR analysis demonstrated a significant reduction in TMUV E gene expression in cells treated with GW4869-exosomes (Figure 5G), confirming a substantial decline in their infectivity.

To investigate the immunomodulatory role of exosomes, recipient cells were primed with exosomes purified from TMUV-infected HEK293 cells (24 hpi) prior to TMUV challenge. Results showed that this pre-exposure to TMUV-exosomes significantly suppressed the subsequent expression of key antiviral immune genes, including *IFN-β, IFIT-3*, *ISG56*, and *TNF* (*P* < 0.01) (Figure 5H). This dampened host defense response consequently facilitated enhanced viral replication.

### TMUV NS4A promotes the exosome-mediated transmission pathway by upregulating Rab27a expression

**Figure 6:**
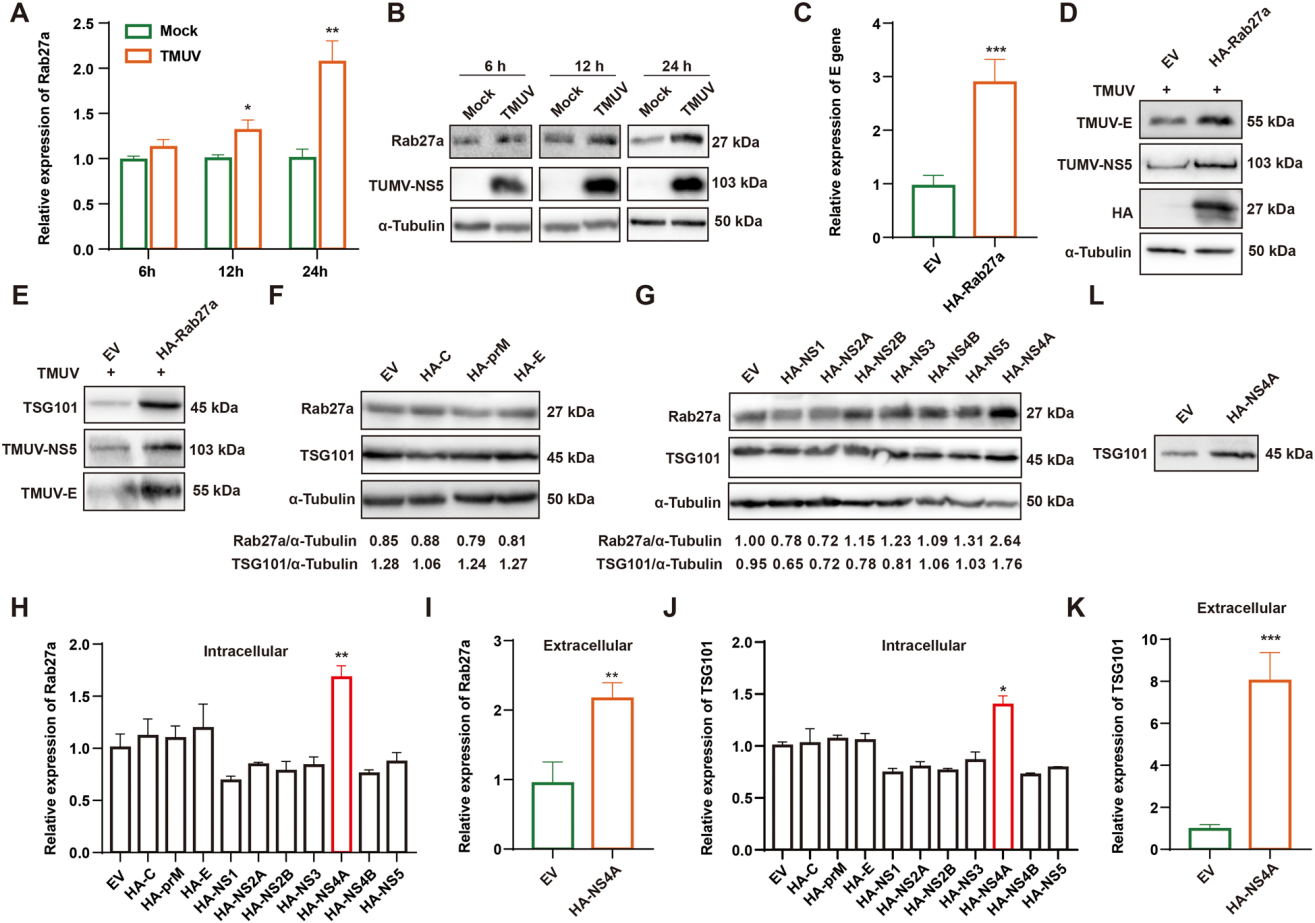
**TMUV NS4A governs the exosome-mediated transmission pathway by contributing Rab27a induction**. **A** qPCR analysis of Rab27a mRNA expression in HEK293 cells infected with TMUV at the indicated periods. **B** Immunoblotting analysis of Rab27a protein levels and TMUV NS5 expression in HEK293 cells infected with TMUV (MOI = 1) at the indicated periods. **C** qPCR analysis of TMUV E mRNA expression in HEK293 cells transfected with empty vector (EV) or HA-Rab27a plasmid for 24 h followed by TMUV infection for 24 h. **D** Immunoblotting analysis of TMUV E and NS5 protein levels in HEK293 cells transfected with empty vector or HA-Rab27a plasmid for 24 h followed by TMUV infection for 24 h. **E** Immunoblotting analysis of TSG101, NS5, and E protein levels in TMUV-exosomes isolated from EV- or HA-Rab27a-transfected, TMUV-infected HEK293 cells. **F** Immunoblotting analysis of Rab27a and TSG101 protein expression in whole-cell lysates of HEK293 cells transfected with EV or TMUV structural protein (C, prM, E)-expressing plasmids for 48 h. **G** Immunoblotting analysis of Rab27a and TSG101 protein expression in whole-cell lysates of HEK293 cells transfected with EV or TMUV nonstructural protein (NS1–NS5)-expressing plasmids for 48 h. **H** qPCR analysis of Rab27a mRNA expression in whole-cell lysates of HEK293 cells transfected with EV or TMUV protein-expressing plasmid for 24 h. **I** qPCR analysis was performed to detect Rab27a mRNA expression in the supernatant of HEK293 cells transfected with either EV or TMUV NS4A expression plasmid for 48 h. **J** qPCR analysis of TSG101 mRNA expression in whole-cell lysates of HEK293 cells transfected with EV or TMUV protein-expressing plasmid for 24 h. **K** qPCR analysis was performed to detect TSG101 mRNA expression in the supernatant of HEK293 cells transfected with either EV or TMUV NS4A expression plasmid for 48 h. **L** Immunoblotting analysis of TSG101 expression in exosomes isolated from HEK293 cells transfected with EV or HA-NS4A plasmid for 24 h.

To elucidate the molecular mechanisms governing exosome secretion, this study focused on Rab27a—a key regulator of exosome trafficking—previously identified in our label-free quantitative proteomics data. We first assessed Rab27a expression in HEK293 cells following TMUV infection. RT-qPCR and Western blotting analyses at multiple time points revealed that TMUV infection significantly upregulated Rab27a at both the transcriptional and protein levels (Figure 6A). To further investigate the functional role of Rab27a, we constructed a eukaryotic expression plasmid, pCAGGS-Rab27a, and transfected it into HEK293 cells. Upon TMUV infection 24 h post-transfection, overexpression of Rab27a markedly enhanced TMUV replication, as evidenced by increased expression of the E and NS5 proteins and elevated E gene expression (Figure 6B). We next examined whether Rab27a influences exosome biogenesis and viral cargo packaging. Exosomes isolated from Rab27a-overexpressing and TMUV-infected cells showed elevated levels of the exosomal marker TSG101, as well as increased incorporation of TMUV E and NS5 proteins (Figure 6C). These results indicate that Rab27a enhances both exosome secretion and the loading of viral proteins into exosomes. Given that TMUV infection promotes exosome release, we sought to identify the specific viral protein responsible. Among the three structural and seven nonstructural proteins of TMUV tested, overexpression of NS4A significantly increased the expression of Rab27a and TSG101 at both the messenger RNA (mRNA) and protein levels (Figure 6D–I). Furthermore, exosomes isolated from NS4A-overexpressing cells exhibited increased TSG101 expression (Figure 6J), confirming that TMUV NS4A enhances exosome secretion. In summary, our findings demonstrate that TMUV NS4A upregulates Rab27a and TSG101 expression, thereby promoting exosome release and enhancing the packaging of viral proteins into exosomes. This mechanism facilitates cell-to-cell transmission of TMUV via exosomes and highlights a novel role for NS4A in viral spread.

## Discussion

Exosomes, a predominant subclass of extracellular vesicles delimited by a lipid bilayer, are constitutively released by nearly all cell types and are ubiquitously present in various biological fluids [28]. The role of exosomes in mediating intercellular communication was first brought to light in 1996, when Raposo and colleagues demonstrated that exosomes derived from human and murine B lymphocytes could elicit major histocompatibility complex class II-restricted T-cell responses and modulate immune cell functions [29]. In recent years, accumulating evidence has underscored the capacity of exosomes and their molecular cargo to interact with recipient cells, thereby facilitating interscellular exchange of bioactive molecules and playing pivotal roles in regulating diverse cellular processes [30–32].

The overlapping size distribution of exosomes and viruses poses a major challenge in obtaining pure exosome preparations from virus-infected cell cultures. Standard isolation techniques—such as ultracentrifugation, polymer-based precipitation, ultrafiltration, size-exclusion chromatography, and related chromatographic approaches—often yield mixed populations of exosomes and viral particles rather than complete separation [33]. A key distinguishing feature of exosomes is the presence of specific surface markers, including CD63, CD81, and CD9, which are not found on viral particles. This characteristic enables the use of immunoaffinity-based strategies with antibodies targeting these markers for selective exosome isolation [34]. In this study, we employed a multistep purification protocol: differential centrifugation was first applied to remove dead cells, debris, and large vesicles from culture supernatants. This was followed by filtration through a 0.22 μm membrane to exclude vesicles larger than 200 nm, thereby concentrating exosomes within the typical 30–200 nm size range. Exosomes were then pelleted by ultracentrifugation, and the resulting exosome–virus mixtures were further purified using CD63-conjugated immunomagnetic beads to remove contaminating viral particles, yielding highly pure exosome preparations. While nanoparticle tracking analysis (NTA) is widely used for exosome size characterization, it has several limitations. In recent years, NanoFCM has emerged as a promising alternative due to its high sensitivity, wide dynamic size range, and rapid analysis capability. Here, NanoFCM was used to determine the size distribution and concentration of purified exosomes, confirming that the isolated particles fell within the expected 30–200 nm exosomal size range. We also investigated the effect of TMUV infection on exosome biogenesis and physical properties. Infection significantly enhanced exosome secretion, as indicated by increased particle concentrations and elevated expression of the exosomal marker TSG101. To ensure the physiological relevance of these findings, we validated this model in both human HEK293 cells and avian-derived HD11 cells. Our comparative analysis demonstrated that viral replication kinetics and exosome characteristics remained highly consistent across both cell types (data not shown), justifying the use of HEK293 cells for subsequent mechanistic dissections. Interestingly, however, TMUV infection resulted in a noticeable reduction in exosome diameter in HEK293 cells—a finding that contrasts with reports on other flaviviruses, which often lead to an increase in exosome size [35]. Although the mechanisms underlying virus-induced changes in exosome dimensions remain poorly understood, we hypothesize that the observed size reduction may reflect alterations in protein composition and cargo loading within exosomes during TMUV infection.

Viruses rely exclusively on host cells to complete their replication cycle, with essential processes—including entry, replication, translation, assembly, and egress—dependent on the cellular endomembrane system. This dependency is particularly pronounced for enveloped viruses, whose entry and budding mechanisms closely resemble the biogenesis and release pathways of exosomes. Growing evidence indicates that exosomes derived from cells infected with viruses carry viral nucleic acids, ranging from full-length genomic RNA to partial transcripts, highlighting the selective packaging of viral components into these vesicles [36–40]. In line with these observations, we found that exosomes from TMUV-infected cells contain the full-length viral genome, which can either direct viral replication or be translated into viral proteins within recipient cells, thereby facilitating intercellular viral dissemination. Proteomic profiling further revealed that TMUV-exosomes selectively incorporate the viral C, prM, E, NS1, NS2B, NS4B, and NS5 proteins, while excluding NS2A, NS3, and NS4A. This specificity suggests a regulated packaging process, potentially mediated by the ESCRT machinery involved in exosome biogenesis [41]. Upon internalization by naive cells, these exosomes deliver their cargo into the cytoplasm, establishing a productive infection. Encapsulation within a lipid bilayer shields viral nucleic acids and proteins from immune detection and enzymatic degradation, thereby enhancing the efficiency of intercellular transmission. LC–MS/MS analysis identified a total of 5360 host proteins in exosomal fractions—3211 in mock-derived and 2149 in TMUV-derived exosomes—indicating a reduction in proteomic diversity following infection. Among these, 1106 proteins were differentially expressed, with 52 upregulated and 130 downregulated. These proteins are implicated in critical cellular processes such as endocytosis, vesicular trafficking, viral infection, and autophagy, reflecting TMUV-induced remodeling of the host proteome and its selective loading into exosomes. Notably, exosome biogenesis and trafficking factors, including CHMP4B, VPS4B, and TSG101, were significantly upregulated, and Rab27a was uniquely detected in TMUV-exosomes—consistent with enhanced exosome release during infection. Additionally, viral-infection-related host proteins such as HSPA5, DDX1, and ATP6V1B2 were markedly elevated. These molecules may serve as viral receptors or cofactors [42–44], with ATP6V1B2—a vacuolar proton pump—contributing to endosomal acidification and viral replication [36]. Autophagy-related proteins, including ATP6V1B2, ATP6V1A, ATG3, and ATG7, were also upregulated or exclusively detected in TMUV-exosomes. Collectively, these findings demonstrate that TMUV infection reprograms the host proteome and promotes selective packaging of viral and host components into exosomes. These altered exosomes may modulate recipient cell functions and facilitate viral spread, illustrating a sophisticated mechanism of non-lytic viral dissemination.

Exosomes can be internalized by recipient cells through multiple endocytic pathways—including clathrin-mediated endocytosis, macropinocytosis, caveolin-dependent uptake, and lipid raft-mediated entry—entering via the endosomal system before releasing their contents into the cytoplasm to modulate cellular functions [45]. Alternatively, exosomes may exert effects without full internalization, by directly engaging surface receptors and activating signaling cascades [46]. Our exosome uptake assays demonstrated that TMUV-exosomes are actively internalized by recipient cells, with the intracellular fluorescence signal intensifying over time, indicating that cellular internalization is a prerequisite for their infectivity. When cells were treated with equivalent amounts of exosomes or live virus, the viral prM protein was detectable as early as 36 h postinfection with cell-derived TMUV, whereas it was only observed at 48 h following treatment with TMUV-exosomes. This suggests that, while TMUV-exosomes retain infectivity comparable to live virus, their intracellular replication proceeds at a slower rate. Crucially, our study reveals a novel entry pathway for TMUV-exosomes that differs from other flaviviruses. While exosomes from viruses such as Dengue or Zika predominantly use clathrin-mediated endocytosis or macropinocytosis, we demonstrate that TMUV-exosomes specifically require caveolin-mediated endocytosis for cellular entry. This represents the first report identifying caveolin-mediated endocytosis as the primary route for flavivirus-related exosome uptake. The infectivity of exosomes varies considerably among different viruses. For instance, exosomes derived from EV71-infected cells exhibit stronger pathogenic effects and higher replication efficiency than their corresponding viral particles [37], whereas the opposite holds true for exosomes from foot-and-mouth disease virus (FMDV)-infected cells [40]. In our time-course infection assays, exosomes collected at 4 and 8 h post-TMUV infection showed no significant effect on viral replication, whereas those harvested at 12 h significantly enhanced TMUV replication, with an even more pronounced effect observed at 24 h. This temporal pattern may reflect the dominant role of innate immune responses during early infection stages, where exosomes could potentially package antiviral components such as interferons, thereby mitigating viral infection [47, 48]. In this study, regardless of whether cells were treated with GW4869 before or after TMUV infection, both viral replication and the packaging of viral proteins into exosomes were reduced. In studies on Zika virus (ZIKV)-exosomes, pretreatment with GW4869 did not affect viral attachment to the cell membrane or the expression of the ZIKV receptor AXL, indicating that GW4869 inhibits viral replication through mechanisms unrelated to viral adsorption or receptor expression [49]. Interestingly, ZIKV infection upregulates neutral sphingomyelinase activity both intracellularly and in exosomes, which correlates with enhanced viral replication—though the underlying mechanism remains unclear [50]. In contrast, GW4869 does not affect Porcine reproductive and respiratory syndrome virus (PRRSV) replication [34]. Therefore, the mechanisms by which GW4869 inhibits TMUV replication and reduces viral content in exosomes warrant further investigation.

LC–MS/MS analysis revealed the presence of Rab27a protein in exosomes derived from TMUV-infected cells. As a member of the Rab GTPase family, Rab27a plays a critical role in mediating the fusion of multivesicular bodies (MVBs) with the plasma membrane [46]. Studies have shown that Rab27a also functions in the regulation of viral infection processes [51–54]. In this study, both protein and gene expression levels of Rab27a were significantly upregulated following TMUV infection. Conversely, overexpression of Rab27a enhanced viral replication, indicating a mutually reinforcing relationship between Rab27a expression and TMUV replication. Consistent with previous findings, Rab27a overexpression in HEK293 cells led to a significant increase in exosome production. Furthermore, Rab27a overexpression followed by TMUV infection resulted in markedly elevated levels of TMUV E and NS5 proteins in exosomes. This may be attributed to the use of MVB and recycling endosome membranes by enveloped viruses for their encapsulation, combined with the involvement of Rab27a in viral capsid formation, thereby accelerating virion assembly [55, 56]. As viral proteins are selectively packaged into exosomes via the endosomal pathway, increased Rab27a expression enhances MVB docking and release at the plasma membrane, ultimately leading to increased secretion of exosomes carrying TMUV components. Our results demonstrate that TMUV infection promotes exosome release, and that TMUV-exosomes in turn enhance TMUV replication, with Rab27a playing a key role in this process. Unlike previous studies that implicated structural proteins or NS1 in exosome incorporation, we identify the nonstructural protein NS4A as the principal regulator of TMUV-exosome biogenesis. Furthermore, our findings show that TMUV induces the transcriptional upregulation of Rab27a through NS4A, creating a specific feed forward loop that amplifies viral dissemination. This molecular axis provides a distinct mechanistic insight into how avian flaviviruses hijack the host secretory machinery.

To identify which TMUV protein is responsible for promoting exosome release, we transfected HEK293 cells with each of the ten TMUV proteins and examined changes in the expression levels of Rab27a and TSG101 both intracellularly and extracellularly. The results showed that NS4A protein significantly increased the expression of Rab27a and TSG101 inside and outside the cells, and also markedly enhanced exosome secretion, indicating that TMUV NS4A plays an important role in promoting exosome release. NS4A, one of the nonstructural proteins of TMUV, is primarily involved in viral replication, assembly, and autophagy. The processes of TMUV replication and assembly are closely associated with the replication complex, in which NS4A plays a key role [57]. Studies have shown that NS4A, along with NS4B, NS2A, and the NS1 dimer located in the endoplasmic reticulum lumen, can induce negative membrane curvature, promote membrane bending, and facilitate vesicle formation, thereby assisting in viral genome replication. In this process, the ESCRT machinery also contributes to the formation of vesicle packets [58]. During dengue virus (DENV) entry into host cells, the integrity of microfilaments—specifically, the cytoskeleton—is required. Vimentin interacts with NS4A to regulate the formation of the replication complex, thereby promoting viral replication [59]. Additionally, in DENV and ZIKV infections, NS4A can induce autophagy [60, 61]. Proteomic analysis of TMUV-exosomes did not detect TMUV NS4A protein within the exosomes. Previous studies have noted that, while NS2B and NS4B proteins are readily detectable in exosomes from flavivirus-infected cells, NS4A is rarely observed and is only detected in certain flavivirus infections. The absence of NS4A in TMUV-exosomes may be attributed to this phenomenon. While our data demonstrate that NS4A overexpression consistently upregulates Rab27a expression and that Rab27a enhances exosome-mediated viral transmission, direct causal evidence, such as Rab27a knockdown or NS4A mutant analysis, is needed to definitively establish that NS4A directly drives Rab27a transcription. Addressing these limitations in future work will provide a more comprehensive understanding of the intricate interplay between TMUV NS4A, the host exosomal machinery, and viral transmission, potentially paving the way for novel antiviral strategies.

## Supplementary Information

**Additional file 1. Complete primer sequences of TMUV.**

**Additional file 2. Quantitative real-time PCR primers.**

## Data Availability

Data are provided within the manuscript or supplementary information files.
