# Supplementary material for: Exosomes mediate intercellular transmission of Tembusu virus via the NS4A-Rab27a axis
Source: Vet Res. 2026 Aug 9;57:144. doi: 10.1186/s13567-026-01772-4 (PMC13453093; doi:10.1186/s13567-026-01772-4)
Supplement: Supplementary file 1 [file 13567_2026_1772_MOESM1_ESM.docx]

**Additional file 1 Complete primer sequences of TMUV**

| Primers | Sequences (5’-3’) | Fragment length |
| --- | --- | --- |
| 1F | AGAAGTTCATCTGTGTGA | 1147 |
| 1R | TTTCGGTTCATAGCAGTA |  |
| 2F | TGCTATGAACCGAAAGTG | 1174 |
| 2R | TAAATGCTGAGCCGAAAA |  |
| 3F | GTGGCATAGAAGTGGGAGC | 864 |
| 3R | ACTGCGGTGTCACAATCAG |  |
| 4F | GCCCGAAGATGTCAAATAA | 1405 |
| 4R | GTCAATCCCACAGCAGTCA |  |
| 5F | GGGATAGATGTGGTGAGAAT | 1162 |
| 5R | GTAGGTCCAGCACTGTTAGTT |  |
| 6F | TATGGCAAGAGGACTGTTAGG | 929 |
| 6R | CTGTTATTGGCGAGTTGGA |  |
| 7F | CTCCAACTCGCCAATAACA | 1309 |
| 7R | ACCCACCACTAACAAGACAAC |  |
| 8F | CGCTCACAGAATGACAGAA | 1083 |
| 8R | AAATGGCACATAGCAACAG |  |
| 9F | GCTACAACATTTCTGACTCCCTT | 1252 |
| 9R | GCACCCTAACCAAACCACC |  |
| 10F | GAGGTGGTTTGGTTAGGGT | 1105 |
| 10R | ATGAGTGGTTCTGCGACAG |  |
| 11F | CTGTCGCAGAACCACTCAT | 863 |
| 11R | ACACCAGCCATCTTCCCTC |  |
| 12F | GGTGAGTGGATGACTTCTG | 921 |
| 12R | CGAGACTCTGTGTTCTACCAC |  |
