# Supplementary material for: Exosomes mediate intercellular transmission of Tembusu virus via the NS4A-Rab27a axis
Source: Vet Res. 2026 Aug 9;57:144. doi: 10.1186/s13567-026-01772-4 (PMC13453093; doi:10.1186/s13567-026-01772-4)
Supplement: Supplementary file 2 [file 13567_2026_1772_MOESM2_ESM.docx]

**Additional file 2 Primer used in the study**

| Primers | Sequences (5’-3’) |
| --- | --- |
| TMUV-E-F | CGCTGAGATGGAGGATTATGG |
| TMUV-E-R | ACTGATTGTTTGGTGGCGTG |
| IFIT3-F | TCAGAAGTCTAGTCACTTGGGG |
| IFIT3-R | ACACCTTCGCCCTTTCATTTC |
| IFN-β-F | GCTTGGATTCCTACAAAGAAGCA |
| IFN-β-R | ATAGATGGTCAATGCGGCGTC |
| ISG56-F | GCCATCACAGCCACACAGA |
| ISG56-R | TTTCCCCACAGCCTTAGCA |
| TNF-F | CCTCTCTCTAATCAGCCCTCTG |
| TNF-R Rab27a-F | GAGGACCTGGGAGTAGATGAG GGAGAGGTTTCGTAGCTTAACG CCACACAGCACTATATCTGGGT GGAGAGGTTTCGTAGCTTAACG |
| Rab27a -R TSG101-F |  |
| TSG101-R | CCACACAGCACTATATCTGGGT |
| hsa-β-actin-F | CACCATTGGCAATGAGCGGTTC |
| hsa-β-actin-R | AGGTCTTTGCGGATGTCCACGT |
| chicken-GAPDH-F | GCCATCACAGCCACACAGA |
| chicken-GAPDH-R | TTTCCCCACAGCCTTAGCA |
